# Assessment of Selection Criteria and Influencing Factors in a Plastic Surgery Residency Program in Qatar: Perspectives of Program Directors and Residents

**DOI:** 10.1093/asjof/ojag003

**Published:** 2026-01-09

**Authors:** Mohamed Badie Ahmed, Fatima Saoud Al-Mohannadi, Mohammad Abu Orabi Al-Adwan, Hamad M Al Jaber, Abeer Alsherawi

## Abstract

Plastic surgery residency is highly competitive, with program directors (PDs) using defined criteria for applicant selection. International evidence shows multiple factors influence both leadership decisions and applicant preferences. In Qatar, Accreditation Council for Graduate Medical Education—International (ACGME-I) accreditation and the new Qatari Board shape residency training, yet no local data examine whether directors’ selection criteria align with residents’ program choices or how accreditation and certification impact these decisions. The authors aimed to identify the selection criteria prioritized by current and former PDs and associate PDs (APDs), and to explore the key factors influencing residents’ decisions to join the Qatar Plastic Surgery Residency Program. A cross-sectional study using structured, anonymous online questionnaires was conducted. Surveys assessed demographics, selection priorities, program choice factors, and perceptions of ACGME-I accreditation and Qatari Board certification. Data were analyzed descriptively, comparatively, and thematically. Six PDs/APDs participated. Grade point average and previous plastic surgery experience were the most influential selection criteria, whereas conference presentations and postgraduate degrees were least important. Directors also valued strong clinical rotation reputations, being a fresh graduate, passing licensing examinations, and previous electives; basic-science research and gender were less influential. Nineteen residents responded. ACGME-I accreditation was the strongest factor in program choice, followed by surgical exposure, structured teaching, case diversity, and mentorship. Directors and residents aligned in valuing academic performance and high-quality clinical training, whereas accreditation and certification were key determinants of program choice. These findings provide the first local evidence to guide recruitment strategies and support ongoing development of plastic surgery training in Qatar.

Residency training is a crucial stage in postgraduate medical education, bridging medical school and independent clinical practice. During this period, physicians learn advanced clinical and surgical skills and develop the professionalism required for safe and effective patient care. Because of its impact on future healthcare quality, the residency selection process plays a crucial role in identifying candidates who will excel both clinically and professionally. Program directors (PDs) and associate PDs (APDs) typically assess candidates based on academic performance, clinical experience, research productivity, interviews, and recommendation letters. However, the emphasis placed on these factors varies across institutions and countries.

Residency selection across medical specialties considers both objective and subjective factors. Although academic scores, grades, and research remain important, personal qualities such as professionalism, communication, teamwork, and resilience are increasingly emphasized. It has been reported that letters of recommendation, valued by 86% of PDs with an average rating of 4.2/5, along with perceived commitment to the specialty, including audition or previous clinical rotations, were key factors.^1^ It has similarly been noted that applicant attitude and professionalism were most important for both surgical and nonsurgical directors in Saudi Arabia, whereas letters of recommendation and research varied in significance.^2^

Plastic surgery residency is one of the most competitive and demanding training pathways worldwide. Plastic surgery, with its blend of artistry, innovation, and technical precision, has a distinctive selection process. It has been observed that letters of recommendation from recognized sources were highly important, often ranked above other metrics.^3^ From the applicant's perspective, it has been noted that resident happiness, high operative volume, and strong faculty mentorship strongly influenced program rankings.^4^ Together, these findings show that plastic surgery selection depends on both academic performance and interpersonal qualities such as motivation and collaboration.

Qatar has emerged as a regional leader in medical education, with Hamad Medical Corporation (HMC) at the forefront. The Plastic Surgery Residency Program at HMC, accredited by the Accreditation Council for Graduate Medical Education—International (ACGME-I), demonstrates the country's commitment to global training standards.^5^ Accreditation has strengthened postgraduate education by improving curricula, faculty development, and the learning environment, leading to greater trainee autonomy, better teaching, enhanced social support, and higher work engagement. Structured roles, mentorship, and clear responsibilities were key contributors, underscoring the value of accreditation in advancing medical training.^6^ In addition, the recent establishment of the Qatari Board of Medical Specialties-Plastic Surgery (QBMS-PS) further introduced a national certification framework that aligns local training outcomes with international best practices.

Despite these significant developments, there is currently no published data evaluating the factors that influence applicant acceptance from the PDs/APDs’ perspective, nor what motivates residents to choose this program. It also remains unclear whether key elements such as ACGME-I accreditation and QBMS-PS impact their decision making. This study addresses this gap by exploring the criteria that PDs/APDs in Qatar prioritize when selecting residents for the plastic surgery training program and the factors that influence residents’ decisions to choose the program. It also examines how ACGME-I accreditation and QBMS-PS certification affect both program leadership perspectives and applicant preferences. Understanding the alignment between selection criteria and applicant motivations is essential for improving recruitment strategies, refining program objectives, and ensuring that the residency program meets institutional and trainee goals.

## METHODS

### Study Design

This cross-sectional, survey-based descriptive study was conducted to assess the selection criteria utilized by Plastic Surgery PDs and APDs in Qatar and to explore the factors influencing residents’ decisions to join the Qatar Plastic Surgery Residency Program. The study also examined perceptions regarding the influence of ACGME-I accreditation and the recently established QBMS-PS on selection processes and applicant preferences. No clinical interventions or experimental treatments were involved. Data were collected through structured, anonymous online questionnaires distributed to 2 target groups: PDs/APDs and current residents.

### Study Population

The study was conducted within the framework of the HMC residency training system and targeted 2 distinct participant groups: PDs/APDs, and current residents of the Qatar Plastic Surgery Residency Program. The PD/APD group included all current and former individuals who have served in these roles since the program's establishment, ensuring comprehensive representation of historical and current selection practices. The resident group comprised all trainees from postgraduate year (PGY) 1 to PGY 6 enrolled during the study period, providing insight into factors influencing their decision to join the program and their perceptions of ACGME-I accreditation and QBMS-PS certification. Participation was voluntary, with inclusion limited to those who completed the online survey. The study involved no clinical interventions or patient-related activities, and data were collected electronically through institutional email channels.

### Sample Size

Given the small and well-defined population of both PDs/APDs and residents within the Qatar Plastic Surgery Residency Program, a total population sampling approach was adopted. This method ensured complete representation and enhanced the reliability and relevance of the findings for institutional and national interpretation.

### Ethical Considerations

All participants provided informed consent before survey completion, and no identifying information was collected to maintain confidentiality. The manuscript is approved by the Hamad Medical Corporation—Medical Research Center with reference number: MRC-01-25-894.

### Statistical Analysis

Descriptive statistics were used to summarize demographic and categorical variables. Frequencies and percentages were reported for categorical data such as gender, nationality, PGY, and survey responses, whereas means and standard deviations were used for continuous variables. For PD and APD responses, proportions were calculated for each binary (yes/no) question to determine the relative importance of candidate selection criteria, including academic background, examination performance, and professional attributes. Mean and median years of experience were computed to describe leadership tenure within the Qatar Plastic Surgery Residency Program.

For resident survey data, descriptive analyses were performed to assess perceptions of training quality, influential program factors, and attitudes toward ACGME-I accreditation and QBMS-PS certification. The Likert-scale questions (ranging from “not important” to “extremely important”) were analyzed using frequency distributions to identify the most and least valued program attributes. Multiple-response questions (eg, perceived areas improved by ACGME-I) were analyzed by calculating the proportion of selections relative to total responses.

Qualitative responses were analyzed thematically using an inductive approach. Recurring themes were identified, coded, and categorized into key domains such as mentorship, surgical exposure, accreditation impact, and institutional reputation. Representative quotes were extracted to support thematic interpretations. All statistical analyses were descriptive in nature because of the small and defined study population. Results were presented in tables and figures to illustrate key findings regarding residency program perceptions, leadership perspectives, and the influence of accreditation systems on training and career outcomes.

## RESULTS

### Program Directors/Associate Program Directors Section

A total of 6 PDs and APDs were included in the analysis, representing all individuals who have held this position since the program's inception. Among the 6 participants, there was an equal gender distribution, with 3 males (50%) and 3 females (50%). Half of the respondents were current PDs/APDs, whereas the remaining 3 were former directors. The participants’ duration of experience in these roles ranged from 1 to 10 years, with a mean of 4.8 years and a median of 4 years.

**Figure 1.**
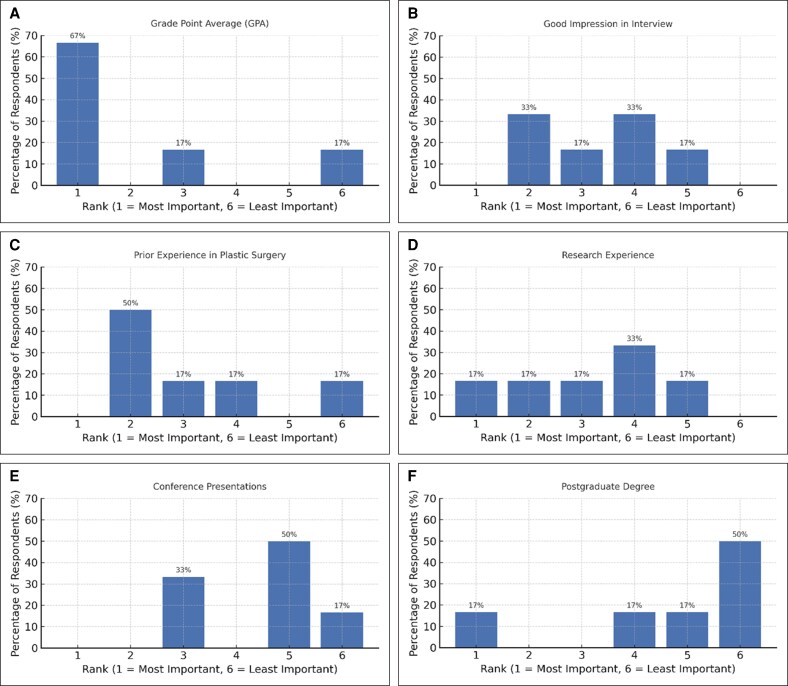
The rank of the following factors on a scale from 1 to 6, with 1 being the most important and 6 the least important. (A) Grade point average. (B) Good impression in interview. (C) Previous experience in plastic surgery. (D) Research experience. (E) Conference presentations. (F) Postgraduate degree.

The ranking question assessing the importance of various applicant factors on a scale from 1 (most important) to 6 (least important) revealed that grade point average (GPA) was considered the most influential criterion, with 67% of PDs ranking it first. Previous experience in plastic surgery followed, ranked as the second most important factor by 50% of respondents. Good impression during the interview received mixed evaluations, primarily distributed between Ranks 2 and 4 (33% each). Research experience demonstrated the greatest variability, with no clear consensus among respondents. In contrast, conference presentations and postgraduate degrees were consistently rated lower in importance, with 67% of PDs ranking each in the lower importance categories (Ranks 5 and 6; Figure 1).

Furthermore, when asked about how recommendation letters, research activity, and previous experience in plastic surgery influence their selection decisions, PDs most frequently valued the reputation of the recommending person and the quality of publications as key indicators of candidate strength. Nearly, all respondents emphasized previous elective experience within the department as a critical selection factor, whereas elements such as publishing in prestigious journals and the number of recommendation letters were considered less influential (Table 1).

**Table 1.** Program Directors’ Priorities in Applicant Evaluation (*n* = 6)

Domain	Selection factor	Frequency (*n*)	%
Recommendation letters^a^	Reputation of the recommending person	5	41.7
	Quality of language and content of the recommendation	2	16.7
	Recommendation from a program director	2	16.7
	Number of recommendation letters	1	8.3
Previous experience in plastic surgery	Candidate worked/took elective in the department	5	83.3
	Elective with a distinguished plastic surgeon	1	16.7
Research activity^a^	Quality of publications (design, execution, writing)	6	50.0
	1 or 2 publications in plastic surgery	3	25.0
	Quantity of publications (regardless of specialty)	2	16.7
	Publishing in prestigious journals	1	8.3

^a^Two choices were allowed for each participant.

In addition, when asked about additional factors influencing applicant selection, all PDs (100%) agreed that being a fresh graduate and the applicant's reputation during clinical rotations or among faculty positively impacted acceptance decisions. A large majority also reported that passing an international licensing examination (eg, United States Medical Licensing Examination [USMLE]) (83.3%), holding awards or honors (83.3%), and demonstrable knowledge in plastic surgery (83.3%) increased an applicant's likelihood of selection. The reputation of the applicant's medical school was considered important by two-thirds (66.7%) of respondents. In contrast, research experience in basic sciences (33.3%) and applicant gender (33.3%) were less frequently viewed as influential factors in the selection process (Table 2).

**Table 2.** Program Directors’ Responses Toward Several Applicants’ Factors (*n* = 6)

Question	Yes, *n* (%)	No, *n* (%)
Would you be more impressed by a candidate with research experience in basic sciences compared with clinical research?	2 (33.3)	4 (66.7)
Does passing an international licensing examination (eg, USMLE) increase the likelihood of acceptance?	5 (83.3)	1 (16.7)
Does being a fresh graduate (within the last 2 years) give a candidate a higher chance of acceptance?	6 (100)	0 (0)
Is the gender of applicants a factor in acceptance decisions?	2 (33.3)	4 (66.7)
Does the reputation of the applicant's medical school influence their chance of acceptance?	4 (66.7)	2 (33.3)
Does the reputation of the applicant during clinical rotation or among faculty influence their acceptance?	6 (100)	0 (0)
Are awards or honors important in selecting applicants?	5 (83.3)	1 (16.7)
Does demonstrable knowledge in plastic surgery significantly influence selection?	5 (83.3)	1 (16.7)

USMLE, United States Medical Licensing Examination.

### Residents Section

A total of 19 residents participated in the study, with a mean age of 28.9 ± 2.8 years. The sample included 57.9% males and 42.1% females, with 31.6% Qataris and 68.4% non-Qataris. Residents were distributed across all training levels (PGY-1 to PGY-6), and more than half graduated from international medical schools (Table 3). Most participants learned about the Plastic Surgery Residency Program in Qatar during undergraduate medical school rotations (48%) or through colleagues or seniors (32%). Fewer respondents cited residency information sessions or career fairs (8%), official websites (8%), or social media platforms (4%) as their primary sources of information.

**Table 3.** Demographic Characteristics of Plastic Surgery Residents

Variable	Category/statistic	*n* (%) or mean ± SD
Total participants		19 (100)
Gender	Male	11 (57.9)
	Female	8 (42.1)
Nationality	Qatari	6 (31.6)
	Non-Qatari (Kuwaiti, Omani, Pakistani, Afghan, Palestinian, Jordanian, Egyptian, Iranian)	13 (68.4)
Age (years)	Mean ± SD	28.9 ± 2.8
Current PGY	PGY-1	3 (15.8)
	PGY-2	3 (15.8)
	PGY-3	3 (15.8)
	PGY-4	3 (15.8)
	PGY-5	3 (15.8)
	PGY-6	4 (21.0)
Medical school of graduation	Qatar University—College of Medicine	6 (31.6)
	Weill Cornell Medicine—Qatar	2 (10.5)
	University of Jordan	2 (10.5)
	Other international universities (Otago, RCSI, Sulaiman Alrajhi, Najah National, Cairo, Dubai Medical College, University of Leeds)	9 (47.4)
Year of medical graduation	Range	2017-2025
	Mean (±SD)	2021 ± 2.4

PGY, postgraduate year; RCSI, Royal College of Surgeons in Ireland; SD, standard deviation.

The analysis of factors influencing residents’ decisions to join the program revealed that ACGME-I accreditation (78.9%), surgical case exposure and hands-on experience (73.7%), and structured academic teaching (63.2%) were perceived as the most important elements. Career advancement potential (63.2%), diversity of clinical cases (63.2%), and faculty mentorship (57.9%) were also highly regarded. Conversely, work–life balance, salary, and fellowship opportunities were considered of lower relative importance. Overall, ACGME-I accreditation stood out as the most influential factor shaping residents’ program selection (Table 4).

**Table 4.** Factors Influencing Plastic Surgery Residents Program Choice

Factor	Extremely important (%)	Important (%)	Somewhat important (%)	Neutral (%)	Not important at all (%)
Reputation of the training program in Qatar	57.9	36.8	5.3	0	0
Structured academic teaching	63.2	26.3	10.5	0	0
Surgical case exposure and hands-on experience	73.7	21.1	5.3	0	0
Diversity of clinical cases	63.2	31.6	0	5.3	0
Faculty mentorship and support	57.9	26.3	15.8	0	0
Research opportunities	36.8	36.8	21.1	5.3	0
Fellowship opportunities after graduation	36.8	21.1	26.3	15.8	0
Work–life balance	21.1	36.8	31.6	10.5	0
Career advancement potential	63.2	26.3	10.5	0	0
Recommendations from seniors/mentors	42.1	47.4	5.3	5.3	0
Location and living conditions	52.6	15.8	10.5	15.8	5.3
Salary and financial benefits	26.3	36.8	36.8	0	0
ACGME-I accreditation status	78.9	15.8	0	5.3	0
Qatar board certification and recognition	52.6	26.3	21.1	0	0

When asked further about the influence of ACGME-I accreditation on the program, the results showed that nearly all respondents (94.7%) believed it has improved the overall quality of training, with only one respondent (5.3%) indicating otherwise (Table 5). The most frequently cited areas of improvement included a structured curriculum (94.4%), followed by faculty supervision and mentorship (61.1%) and resident welfare and duty hour regulation (61.1%). Additional enhancements were noted in clinical exposure and surgical experience (50.0%), evaluation and feedback processes (33.3%), and research support (33.3%). Moreover, 94.7% of participants believed that graduating from an ACGME-I accredited program would positively impact their career opportunities, highlighting the perceived long-term value of accreditation in advancing professional development. Residents’ opinions were asked about the QBMS-PS, and the results showed that most participants (63.2%) believed the certification adds value to their training and career, whereas 31.6% were uncertain and 5.3% disagreed. Regarding its importance, the majority rated it as somewhat or very important (94.7%), underscoring its perceived relevance for future career advancement.

**Table 5.** ACGME-I Influence on the Program

Question	Response	Count	%
Do you believe ACGME-I accreditation improved the quality of training?	Yes	18	94.7
	No	1	5.3
If yes, in which areas have ACGME-I accreditation improved the program?^a^			
Structured curriculum		17	94.4
Faculty supervision and mentorship		11	61.1
Resident welfare and duty hour regulation		11	61.1
Clinical exposure and surgical experience		9	50.0
Evaluation and feedback process		6	33.3
Research support		6	33.3
Recognized in the United States		1	5.6
Do you believe graduating from an ACGME-I accredited program will positively impact your career opportunities?	Yes	18	94.7
	Not sure	1	5.3

ACGME-I, Accreditation Council for Graduate Medical Education—International. ^a^Multiple answers allowed.

### Qualitative Results

The thematic analysis of factors influencing residency program selection revealed that respondents most frequently cited faculty mentorship, surgical exposure, and program reputation as the primary determinants of their decision to join the program. Accreditation (ACGME-I), cultural familiarity, and academic opportunities emerged as important secondary considerations. Overall, the responses reflected a thoughtful balance between training quality, hands-on experience, and personal alignment with institutional and cultural values (Supplemental Table 1).

The thematic analysis of residents’ perceptions toward ACGME-I accreditation and QBMS-PS certification showed that most participants viewed ACGME-I as a catalyst for enhancing training quality, global recognition, and professional credibility, whereas the QBMS-PS was valued for its regional legitimacy and potential for career advancement. Both systems were perceived as complementary, integrating international standards with local healthcare priorities. Several respondents also emphasized institutional reputation and future fellowship opportunities as additional advantages (Supplemental Table 2).

## DISCUSSION

This study provides an in-depth analysis of the factors that influence both PDs/APDs and residents during the recruitment process for the plastic surgery residency program in Qatar. By combining quantitative and qualitative methods, it offers a dual perspective on how academic achievement, departmental exposure, program accreditation, and mentorship contribute to recruitment decisions in a rapidly evolving surgical training environment. The findings reveal a strong consensus between PDs/APDs and residents, with both groups placing greater value on academic excellence, structured education, and mentorship than on lifestyle or financial incentives.

Among PDs/APDs, 67% identified GPA as the most important criteria for selection, highlighting the significance of academic performance in applicant evaluation. Comparable results from US surveys show that PDs/APDs prioritize academic measures such as clinical grades and standardized test scores among the top selection criteria.^7,8^ Additionally, ∼70% of PDs/APDs valued previous exposure to plastic surgery within the same department, indicating a preference for candidates already familiar with the department's standards and culture. International findings similarly support that performance during in-house or departmental subinternships allows PDs/APDs to directly assess clinical skills and professionalism. At the same time, familiarity with a program remains advantageous for match success, with approximately one-quarter of matched applicants training at their home institutions.^7,9^ All PDs/APDs regarded professionalism and behavior during rotations as essential for ranking applicants, findings consistent with Saudi and North American surveys.^10,11^ Fresh graduates were also universally preferred based on the belief that recent training enhances clinical recall and adaptability, which aligns with reports from Saudi Arabia, where 77% of PDs/APDs favored applicants who graduated within the previous 2 years.^10^ Research experience, academic awards, and passing international examinations such as the USMLE were viewed positively, whereas basic-science research and gender had lesser impact. These patterns suggest that PDs/APDs value scholarly engagement as evidence of discipline and scientific literacy rather than focusing solely on publication quantity, a trend consistent with global and regional shifts toward quality-based evaluation.^7-10^ This preference aligns with US data showing that publishing ∼10 to 15 papers does not significantly improve match outcomes.^12^ PDs/APDs in this cohort similarly preferred meaningful, well-mentored research over volume, reflecting a culture that values depth and impact rather than quantity.^12,13^

Residents provided insight into applicant priorities, with surgical exposure,73.7%, and structured academic teaching, 63.2%, ranked among the most important factors, highlighting the value of technical experience and organized education.^14^ These priorities are consistent with data from Saudi Arabia, where 72% to 80% of residents valued operative diversity and structured curricula, and with North American surveys showing that 75% to 85% cited operative autonomy, mentorship, and case volume as primary determinants of satisfaction.^4,11^ Other motivators included program reputation and mentorship opportunities, 60%, reflecting a global preference for programs with strong academic identities and supportive faculty. Only a minority considered work–life balance or financial compensation influential, <30%, mirroring US findings where fewer than 25% of applicants cited lifestyle as a deciding factor.^4,11^ Both PDs/APDs and residents repeatedly identified mentorship as a cornerstone of program attractiveness. More than 60% of residents attributed their decision to apply or remain in a program to mentorship quality and faculty approachability. This finding is supported by systematic reviews showing that 65% to 75% of students with formal mentorship pursue their mentor's specialty and by studies linking structured mentorship to greater academic productivity and retention, particularly among women in plastic surgery.^15,16^ The emphasis on mentorship is also linked with early exposure. PDs/APDs ranked previous departmental experience as the second most valuable selection factor, indicating that active engagement within the specialty predicts applicant success. Internationally, 70% to 80% of matched applicants have completed at least 1 plastic surgery rotation, reinforcing the significance of elective opportunities.^7,9^ Institutional reputation and academic environment were also important to resident decision making. Approximately two-thirds, 63%, prioritized structured academic teaching, whereas 60% to 65% cited program reputation and faculty mentorship as decisive factors. These results align with international reports showing that 60% to 70% of applicants consider academic structure and learning environment critical.^4^ In Saudi Arabia, 64% of trainees similarly selected programs based on reputation and advancement opportunities.^11^ ACGME-I accreditation also emerged as one of the most influential factors in residents’ program selection, cited by 78.9% of respondents. Most residents, 94.7%, agreed that accreditation enhanced the quality of training, supervision, and career opportunities. These findings are consistent with regional studies, where up to 96% of trainees identified accreditation as a major factor in choosing a program.^6,17^ Residents described accreditation as a system that promotes structured learning, consistent supervision, and positive resident well-being, as demonstrated in published evaluations from Qatar showing significant improvements in autonomy, feedback, and curriculum integration after ACGME-I implementation.^6^ Likewise, the introduction of QBMS-PS certification was recognized as a key milestone, offering national recognition and alignment with international training standards. Together, the dual framework of ACGME-I and QBMS-PS certification creates a hybrid model that strengthens both global credibility and local relevance. This integrated structure allows programs to meet international standards while addressing national workforce needs, ultimately enhancing reputation, accountability, and the overall quality of postgraduate surgical education.

The overlap between PDs/APDs and resident priorities points to a well-aligned training environment. Both groups emphasize academic excellence, operative exposure, mentorship, and accreditation. However, PDs/APDs tend to place more weight on objective academic measures (GPA, 67%; research, 83%; in-house rotations, 70%), whereas residents focus more on mentorship and operative volume, 70%. This pattern reflects international trends, where PDs/APDs prioritize measurable competence and applicants value experiential quality.^9,11^ Such alignment promotes transparency in recruitment and reduces the risk of mismatched expectations between programs and applicants. Importantly, the open-ended responses in this study added depth to these findings by showing why participants valued certain factors. Residents described how supportive mentorship and a positive program culture shaped their motivation and sense of belonging, whereas PDs/APDs highlighted the importance of professionalism and personality traits that cannot be fully measured through scores. These insights made the results more personal and meaningful, helping connect the quantitative findings to the lived experiences behind them. On the other hand, the quantitative alignment across these measures carries valuable strategic implications. The shared emphasis on GPA and previous departmental exposure highlights the need for consistent evaluation methods, particularly for applicants from diverse educational backgrounds. Providing structured observerships or electives for external candidates during medical school can help ensure fairness. Maintaining ACGME-I standards also remains essential, because most residents recognized accreditation as a key factor in improving training quality and supervision. Likewise, encouraging a research culture that values depth over publication volume helps preserve academic integrity without creating unnecessary pressure. Continued encouragement of faculty development, especially in fair evaluation, constructive feedback, and mentorship, will be vital to sustaining program quality and reputation.

This study provides a unique and valuable reference for this type of data, offering plastic surgery residents and faculty in the country and even in the region important insights that have not been previously documented. It delivers beneficial information and addresses key points related to selection criteria and influencing factors within the residency pathway. However, it is limited by the relatively small sample size of both PDs/APDs and residents compared with larger international programs, which may affect the generalizability of the findings. In addition, the cross-sectional nature of the study highlights the need for longitudinal data and future comparisons between the factors residents identify during selection and the factors that influence their actual clinical practice over time.

## CONCLUSIONS

This study offers the first local assessment of how PDs’ selection priorities align with the factors influencing residents’ decisions to join the Qatar Plastic Surgery Residency Program. Academic performance, previous specialty exposure, and training quality were consistently valued, whereas ACGME-I accreditation and QBMS-PS certification emerged as key determinants of program choice and perceived career advantage. Future work should expand and include additional institutions and incorporate broader outcome measures to validate these findings. It will also be important to follow graduates over time to assess the real-world benefits they gain from QBMS-PS certification and ACGME-I accreditation, and to determine whether these credentials meaningfully influence their career progression. Evaluating long-term resident performance, career trajectories, and the impact of structured observerships and mentorship will further strengthen the evidence base and guide ongoing development of plastic surgery training in Qatar.

## Supplementary Material

ojag003_Supplementary_Data
