# Supplementary material for: Assessment of Selection Criteria and Influencing Factors in a Plastic Surgery Residency Program in Qatar: Perspectives of Program Directors and Residents
Source: Aesthet Surg J Open Forum. 2026 Jan 9;8:ojag003. doi: 10.1093/asjof/ojag003 (PMC12907024; doi:10.1093/asjof/ojag003)
Supplement: ojag003_Supplementary_Data [file ojag003_supplementary_data.zip › Supplemental Table 1.docx]

**Supplemental Table 1. Thematic Analysis of Factors Influencing Residency Program Selection.**

| **Theme** | **Frequency (n = 19)** | **Representative Quotes** | **Summary** |
| --- | --- | --- | --- |
| **1. Faculty expertise and mentorship quality** | 4 | “Faculty expertise – strong mentors can significantly enhance my learning experience.” “Balance of case types and approachability/support of the seniors.” | Faculty guidance and mentorship were seen as central to learning and professional growth, highlighting the importance of trainer quality and accessibility. |
| **2. Surgical case exposure and hands-on experience** | 5 | “High surgical volume means more hands-on experience.” “Huge surgical exposure, great hands-on experience.” | High clinical volume and operative exposure were key motivators, reflecting a strong preference for experience-based training. |
| **3. Program reputation and feedback from peers** | 3 | “Through a colleague I heard greatly about the program.” “Positive feedback from current residents.” | Peer recommendations and resident satisfaction strongly influenced applicants’ decisions. |
| **4. Program structure, curriculum, and accreditation** | 3 | “Structured curriculum and ACGME-I accreditation.” “Program location and accreditation.” | The presence of a structured training framework and international accreditation (ACGME-I) increased program appeal. |
| **5. Cultural and geographic familiarity** | 2 | “Culture of the people, living style in Qatar, GCC country.” “Family working in the country.” | Proximity to home, cultural alignment, and lifestyle factors played a supportive role in decision-making. |
| **6. Research, conferences, and international exposure** | 2 | “Access to internationally trained faculty and visiting professors.” “Opportunities for regional and international conferences and research projects.” | Academic exposure and collaboration opportunities enhanced perceived program value. |
| **7. Institutional reputation and environment** | 2 | “HMC is the only hospital in Qatar providing reconstructive surgery services.” “Non-toxic environment, great elective rotation experience.” | Institutional exclusivity, case diversity, and a positive learning atmosphere were strong attractors. |
| **8. Intrinsic motivation and personal interest** | 2 | “Personal passion for plastic surgery.” “Working in the field you enjoy the most.” | A few respondents highlighted intrinsic motivation and alignment with personal career goals. |
| **9. No additional factors reported** | 2 | “None.” | A minority reported that no additional factors influenced their decision. |
