# Supplementary material for: Assessment of Selection Criteria and Influencing Factors in a Plastic Surgery Residency Program in Qatar: Perspectives of Program Directors and Residents
Source: Aesthet Surg J Open Forum. 2026 Jan 9;8:ojag003. doi: 10.1093/asjof/ojag003 (PMC12907024; doi:10.1093/asjof/ojag003)
Supplement: ojag003_Supplementary_Data [file ojag003_supplementary_data.zip › Supplemental Table 2.docx]

**Supplementary Table 2. Thematic Analysis of Residents’ Perceptions Toward ACGME-I Accreditation and Qatar Board Certification.**

| **Theme** | **Frequency (n = 19)** | **Representative Quotes** | **Summary** |
| --- | --- | --- | --- |
| **1. Enhanced training quality and educational standards** | 6 | “ACGME-I accreditation ensures the curriculum meets internationally recognized standards.” “ACGME-I ensures that the standard of training is maintained throughout the program.” | Residents recognize ACGME-I as a framework that promotes structured education, objective assessment, and consistent quality across programs. |
| **2. Increased global recognition and fellowship opportunities** | 8 | “Has yet to affect me as I am just starting; however, I believe there will be increased chances for fellowships abroad.” “Some centers in the US have ACGME-I as a criterion for acceptance.” | ACGME-I is viewed as a key enabler for international fellowships, academic opportunities, and mobility across global institutions. |
| **3. Regional credibility and career advancement through Qatar Board** | 5 | “Qatar Board strengthens regional career opportunities.” “Being born and raised in the GCC, the Qatari Board is far more distinctive than the Arab Board.” | Respondents highlighted the Qatar Board’s growing influence in regional healthcare systems and its potential to enhance professional standing within the GCC. |
| **4. Balanced local and global standards** | 3 | “The combination creates a balanced environment between global best practices and Qatar’s healthcare realities.” | Participants appreciated the complementarity between ACGME-I’s international standards and the Qatar Board’s local contextualization. |
| **5. Improved resident welfare and fairness** | 1 | “After ACGME-I came, working hours and calls became more fair for the residents.” | A small number noted tangible improvements in duty hours, fairness, and workload balance after ACGME-I implementation. |
| **6. Pride and national contribution** | 1 | “As a Qatari, I feel a real sense of pride and responsibility to contribute to building its reputation for excellence.” | Some viewed participation in the Qatar Board system as a source of national pride and personal contribution to healthcare development. |
| **7. Professional reputation and credibility** | 3 | “ACGME-I and Qatar Board strengthen residency training by maintaining high standards.” “Credibility and recognition of my training and fellowship opportunities.” | Accreditation was strongly associated with program credibility, reputation, and institutional recognition. |
| **8. No or limited current effect** | 1 | “Has yet to affect me as I am just starting.” | A minority indicated minimal personal impact at early training stages but anticipated future benefits. |
